# Mating-Induced Behavioral Changes in Female *Drosophila* Are Modulated by Serotonin

**DOI:** 10.3390/ijms27167070

**Published:** 2026-08-07

**Authors:** Simón Guerra-Ayala, Antonella Cortés-Henott, Paula Amado-Hinojosa, Marcia González-Teuber, Ivana Gajardo, Jorge M. Campusano

**Affiliations:** Facultad de Ciencias Biológicas, Pontificia Universidad Católica de Chile, Santiago 8331150, Chile; simn.guerra@uc.cl (S.G.-A.); amcortes1@uc.cl (A.C.-H.); pjamado@uc.cl (P.A.-H.); mgonzat@uc.cl (M.G.-T.)

**Keywords:** locomotor activity, social interaction, *Drosophila* females, serotonergic system, post-mating behavioral response

## Abstract

Mating influences the physiology of female animals and induces changes in behaviors associated with securing resources for their offspring. Modulatory neurotransmitters are known to adjust behavioral responses of animals according to environmental cues and internal physiological states. In that regard, it has been shown that serotonin (5-HT) modulates some post-mating behavioral features in *Drosophila*. However, it remains unknown whether serotonin regulates post-mating social behavior or responses to food cues in flies, which are among several behaviors that serve to secure their survival and reproduction. Here, we report a behavioral paradigm, the yeast-response test (YRT), for exploring locomotor and social behavioral changes in groups of virgin and mated females using yeast odor as a naturalistic food signal. Using this setup, we found that exposure to yeast odor increases social interactions in mated but not virgin females under non-aversive conditions and that these responses are suppressed at aversive temperatures. Furthermore, chronic impairment of serotonin transporter function reverses the social behavioral response to food odor in mated females, without affecting their olfactory capacity. Whole-brain 5-HT content, as well as histone serotonylation, were unchanged between virgin and mated females, suggesting that 5-HT contribution to these changes is mediated at a circuit level rather than by global neuromodulatory mechanisms. Consistent with this idea, silencing serotonin projection neurons (SPNs) selectively abolished mating-induced changes in social behavior without affecting the locomotor effects. These results provide further support for data in the literature showing that SPNs belong to a discrete circuit underlying social behaviors in female flies. Together, these results support a model in which serotonergic signaling shapes post-mating behavior in *Drosophila* through functionally segregated neural circuits.

## 1. Introduction

Female animals navigate complex environments, integrating multiple sensory cues to locate food sources and mating partners while avoiding threats that may compromise survival and reproduction. They also explore their surroundings to identify suitable feeding sites and protect their offspring [1,2]. In different animals, including female flies, olfactory cues play a central role in food searching and danger avoidance [3,4,5,6,7].

Importantly, mating is known to induce significant changes in female physiology and behavior to support reproductive success [8,9], a phenomenon also observed in *Drosophila*. Early post-mating responses in flies include a marked reduction in receptivity to additional courting males [10]. In contrast, longer-term changes involve shifts in the internal physiological state that influence behaviors such as foraging and oviposition site selection. These adaptations are reflected in altered locomotor activity, learning and memory capacity, dietary preferences, and social interactions [11,12]. However, the signaling mechanisms underlying these post-mating changes remain incompletely understood.

In *Drosophila*, serotonin (5-HT) is a key neuromodulator that regulates a wide range of behaviors, including navigation in complex environments [5], olfactory processing and memory formation [13,14], all of which are critical for locating and remembering food sources. Increasing evidence also implicates serotonin in post-mating behavioral plasticity. For example, the reduced female receptivity that follows mating [15,16] and the enhancement of olfactory long-term memory in mated females [12] both depend on serotonergic signaling. In fact, the reduced receptivity of mated females towards a new courting male depends on the so-called serotonin projection neurons (SPNs), which belong to a discrete neural circuit [15,16]. Importantly, the contribution of serotonin to other post-mating behaviors remains poorly understood. Likewise, it is unknown whether mating-induced behavioral changes arise from global alterations in serotonergic signaling across the brain or from the modulation of specific serotonergic circuits or neuronal populations like SPNs.

Here, we investigated the role of serotonin in mating-dependent changes in locomotion and social interactions in female *Drosophila*. We also examined whether mating alters whole-brain serotonin levels or histone serotonylation, a recently described serotonin-dependent epigenetic signaling mechanism [17]. Our results demonstrate that serotonin is required for the modulation of locomotor and social responses to food odor following mating. However, these behavioral effects are not associated with changes in whole-brain serotonin content or histone serotonylation levels. Instead, they appear to depend, at least in part, on altered activity within a specific serotonergic neuronal population.

## 2. Results

### 2.1. Mating Induces Changes in Locomotor and Social Behaviors in Female Flies

**Figure 1:**
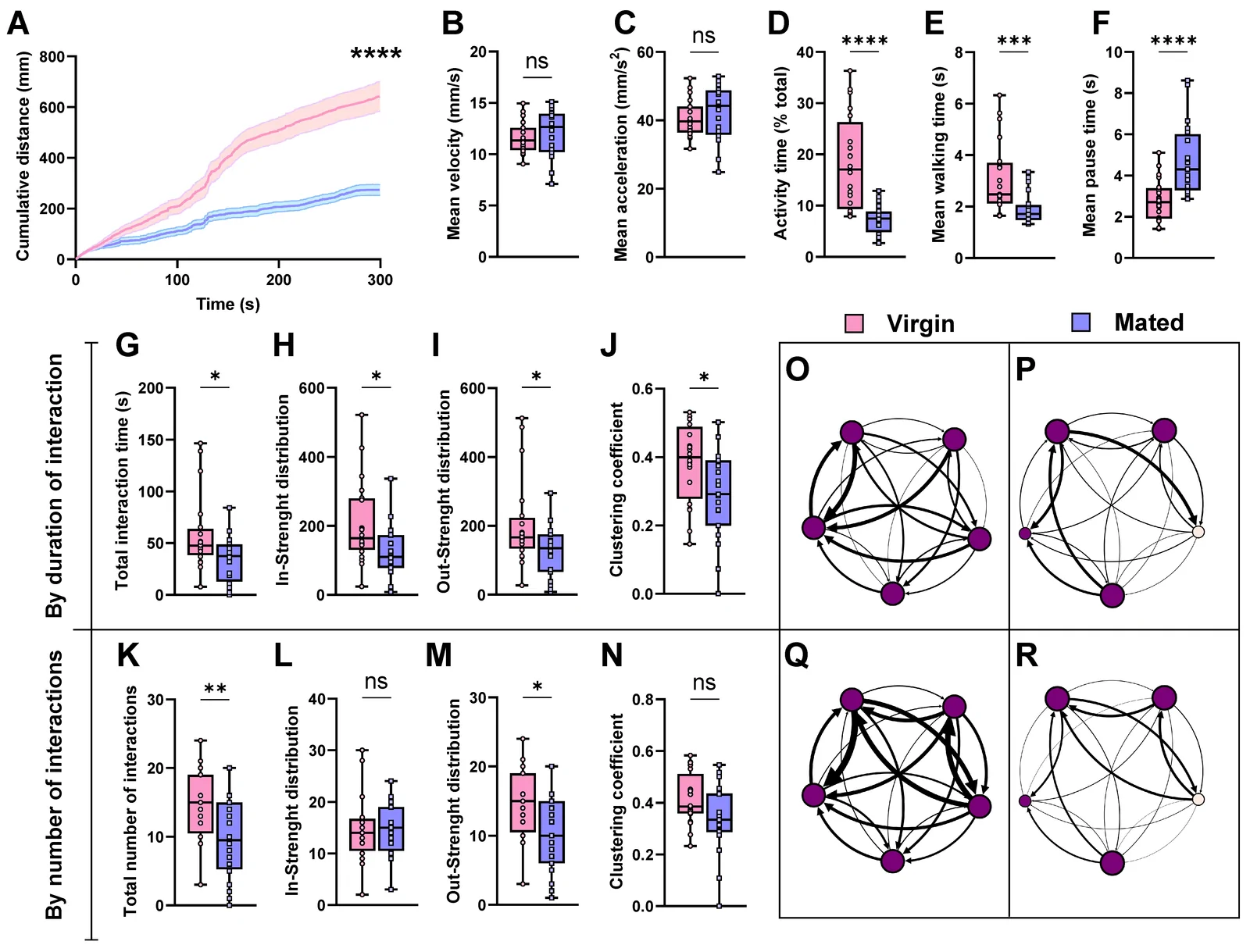
Female flies exhibit decreased locomotion and fly-to-fly interactions post-mating. Behavior was video recorded for 5 min in groups of 4–6 virgin or mated female flies of the CS strain, and their locomotor and fly-to-fly social behaviors were analyzed. (**A**) Cumulative distance traveled by virgin (red) and mated (blue) female flies. Unpaired t test at the final point. (**B**–**F**) Locomotor behavior recorded in virgin and mated female flies and assessed in representative parameters: (**B**) mean velocity, (**C**) mean acceleration, (**D**) activity time, (**E**) mean walking time, (**F**) mean pause time. (**G**–**N**) Representative parameters used to characterize social behavior in virgin and mated female flies. Parameters reflecting the duration of fly-to-fly interactions are shown in (**G**–**J**): (**G**) total interaction time, (**H**) in-strength distribution, (**I**) out-strength distribution, (**J**) clustering coefficient. Parameters reflecting the number of fly-to-fly interactions are shown in (**K**–**N**): (**K**) total number of interactions, (**L**) in-strength distribution, (**M**) out-strength distribution, (**N**) clustering coefficient. An unpaired *t*-test for normally distributed parameters and the Mann–Whitney test for non-normally distributed parameters were used. ns, not significant. *, **, ***, and **** indicate *p* < 0.05, *p* < 0.01, *p* < 0.001, and *p* < 0.0001, respectively. (**O**–**R**) Interaction graphs of representative experiments carried out with groups of five female flies. Nodes represent each fly, and edges denote different interaction criteria, such as the duration (**O**,**P**) or length (**Q**,**R**) of fly-to-fly interaction. Node size represents the degree, and color gradients represent eigenvector centrality. N = 20 flies per condition, from at least three different parentals.

We first investigated whether mating affects locomotor activity or social interactions between female animals. To do this, we video-recorded groups of virgin or mated females of the Canton-S (CS) wild-type strain in a behavioral arena and measured several locomotor parameters. Our results show a reduction in cumulative distance traveled by mated females when compared to virgin females over a 5 min recording period (Figure 1A). When decomposing motor activity of flies in different parameters, we realized that these differences could be explained by higher activity time and walking time of virgin females (Figure 1D,E). At the same time, no differences were detected in mean velocity or the acceleration parameter (Figure 1B,C). Moreover, mated females displayed an increased pause time (Figure 1F), increased pause frequency, and decreased walking frequency (Figure S1). Taken together, this data indicates that mating decreases locomotion, particularly via a reduction in activity parameters, while no change is observed in speed-related parameters.

Next, we studied fly-to-fly interactions among groups of virgin or mated females. This analysis provides a proxy of how social behavior is affected in female animals after mating. We performed a social network analysis and studied parameters that help characterize the duration (Figure 1G–J) and number (Figure 1K–N) of fly-to-fly interactions. Our data show that mating decreases social interaction in groups of mated female flies compared to virgin animals. In fact, the results obtained show a significant reduction in all the parameters used to define the duration of interactions in mated females when compared to virgin counterparts (Figure 1G–J). Our analysis also shows a significant reduction in two parameters that define the number of interactions between mated females: the total number of interactions (Figure 1K) and the out-strength distribution (Figure 1M). Figure 1O–R illustrate social networks in our video recordings and support the idea that the change in social interaction between female virgin and mated flies is explained by differences in the duration of interactions and their frequency, rather than a change in the social network structure, as evidenced by social network parameters (Figure S1). Our data support that mating induces changes in locomotor activity and social interactions in *Drosophila* females.

### 2.2. Food Odor Exposure Induces Mating-Dependent Changes in Social Behavior, Which Are Prevented by Aversive Conditions

It has been shown that animals are attracted to places to raise their offspring through odors that indicate the presence of food [6]. To investigate whether exposure to food odor alters locomotion and social responses and whether this depends on the mating status of flies, we developed a new behavioral paradigm, the yeast-response test (YRT).

**Figure 2:**
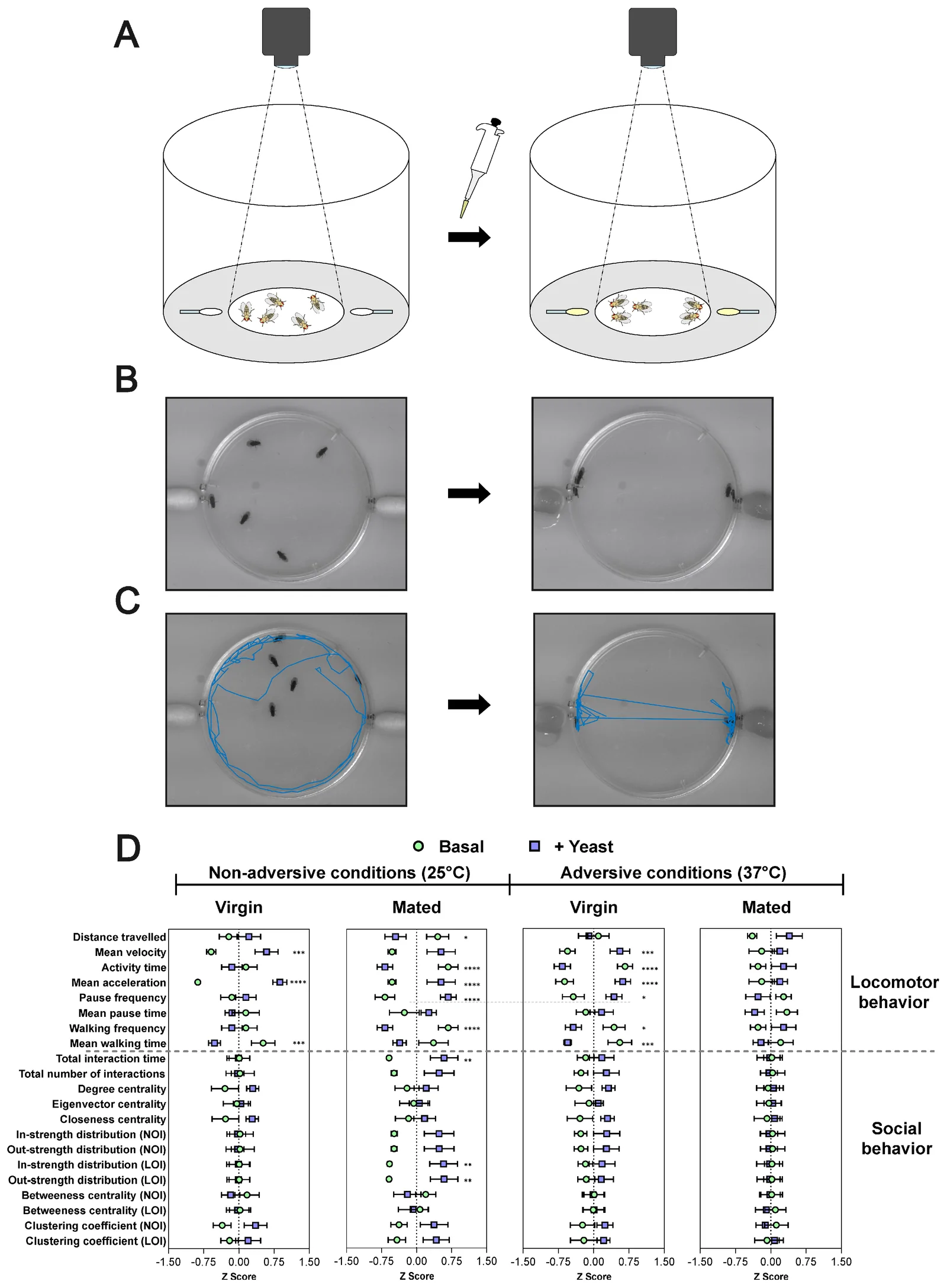
Changes in social behaviors induced by food odor exposure in mated females are not observed at aversive temperatures. (**A**) Schematic illustration of the YRT behavioral setup that shows the conditions without (left panel) and with yeast (right panel). (**B**) Representative images taken from video recordings showing the behavior of flies in the absence or presence of the yeast odor stimulus. (**C**) Representative experiment showing the tracked movement path (blue line) of an individual fly within the group. (**D**) Results obtained for 21 parameters associated with locomotor and social behavior in virgin and mated female flies, when animals are in basal conditions (no odor stimulus) or exposed to food odor, at non-aversive or aversive conditions. Data presented as Z-score normalization. Statistical analysis carried out using unpaired t-tests for normally distributed parameters or Mann–Whitney tests for non-normally distributed parameters with an adjusted *p* value approach using the Holm–Sidak test with correction for multiple comparisons. *, **, ***, and **** represent *p* < 0.05, *p* < 0.01, *p* < 0.001, and *p* < 0.0001. N = 19–20 flies per condition, from at least three different parentals.

In this paradigm, groups of 4–6 flies were allowed to freely move for 5 min (basal conditions), after which they were exposed to yeast odor, a stimulus signaling food, for an additional 5 min. A schematic of this paradigm is shown in Figure 2A, along with representative images of fly recordings and tracking changes following the odor challenge (Figure 2B,C). All strains used in this study showed a significant preference for yeast odor, regardless of the reproductive state, confirming intact olfactory capacity across all experimental groups (Figure S2). Results are presented in Figure 2D as a normalized Z-score for every locomotor and social behavior parameter assessed.

We initially recorded and studied the behavior of virgin and mated female flies in the YRT at 25 °C, in what we call non-aversive conditions. Our results indicate that both virgin and mated female flies show changes in locomotor behavior when exposed to yeast under these conditions (Figure 2D, left panel). However, the quality and characteristics of these changes differ between the two groups in the presence of the food odor. Virgin females showed increases in mean velocity and mean acceleration and a decrease in mean walking time, suggesting faster, more directed movement in response to the odor. In contrast, mated females showed decreased distance traveled, activity time, and walking frequency, with increased pause frequency, indicating an overall reduction in motor output rather than a change in movement speed. These data support that mating not only reduces overall locomotion but also alters the quality of movement in response to food odor.

On the other hand, we detected no changes in social behavior in virgin females exposed to yeast odor when assessed under non-aversive conditions (Figure 2D, left panel). On the contrary, mated flies showed increases in total interaction time, as reflected by the specific parameters in-strength and out-strength distributions according to interaction length (LOI; Figure 2D, second panel). This supports that mated females increase social interactions in response to food odor. Overall, these results suggest that the mating status determines the social behavioral response to food odor in female flies.

We then assessed the YRT at 37 °C, a very high temperature for flies [18,19]. We aimed at assessing whether aversive, stressful conditions affect the locomotor and social behavioral responses to food odor in virgin and mated females (Figure 2D, third and fourth panels). We named this condition aversive. Virgin female flies exposed to yeast odor at 37 °C showed increases in mean velocity and mean acceleration and a reduction in mean walking time, similar to what was observed at non-aversive conditions. However, additional locomotor parameters changed at high temperature, including increased pause frequency and decreased activity time and walking frequency, suggesting that aversive conditions partially alter the quality of movement in virgin females without abolishing the locomotor response induced by the food odor. Importantly, the changes in locomotor and social behavior observed in mated females at non-aversive conditions were not detected when the YRT was performed at 37 °C. Overall, these results support the idea that mating-dependent locomotor and social responses observed in mated females exposed to the food odor are suppressed by aversive environmental conditions. Importantly, the locomotor response observed in virgin females exposed to the food odor is largely preserved even in the presence of the aversive conditions.

### 2.3. The Serotonergic System Modulates Locomotor and Social Responses in a Mating-Dependent Manner

It has been previously shown that 5-HT influences two post-mating behavioral responses in flies: female receptivity to courting males and egg laying [15,16]. Given the well-documented role of serotonergic signaling in regulating baseline locomotion and social dynamics in flies [20,21,22,23], we hypothesized that this amine could also mediate post-mating changes in these behaviors. To assess this, we decided to use a previously characterized hypomorph mutant of the 5-HT transporter (*dSerT*), *dSerT^MI02578^*, as a genetic tool that induces global, chronic changes in serotonergic signaling in flies [21]. The results obtained with this mutant strain were compared to those obtained in its genetic control strain, *w^1118^*.

**Figure 3:**
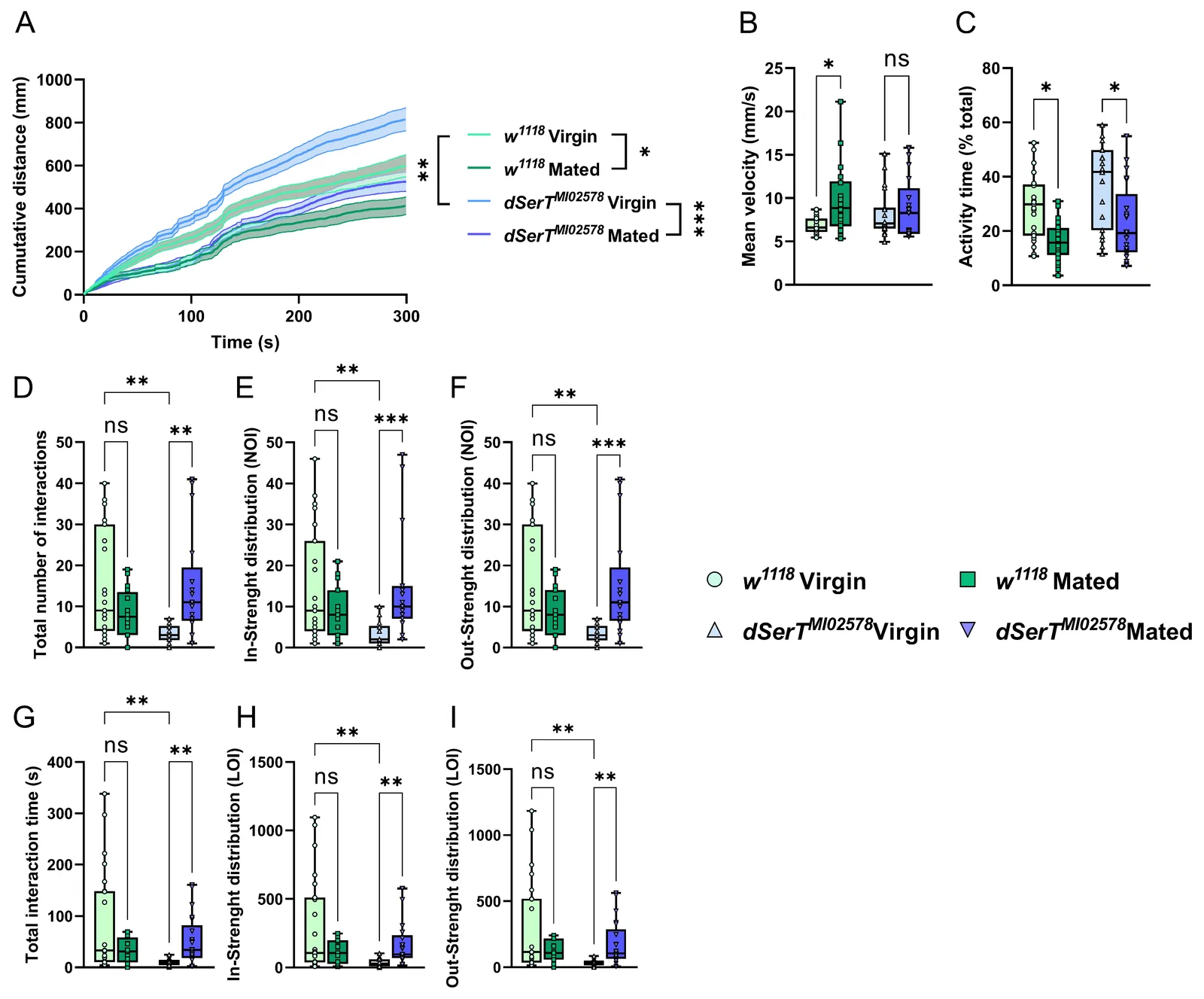
Alteration in serotonergic signaling modifies responses in locomotion and social interactions in female flies post-mating. (**A**) Differences between virgin and mated female flies of the *w^1118^* and *dSerT^MI02578^* strains in the cumulative distance traveled parameter. One-way ANOVA followed by the Holm–Sidak test at final points was used. (**B**,**C**) Changes due to mating in *w^1118^* and *dSerT^MI02578^* strains in (**B**) mean velocity and (**C**) activity time, as representative locomotor parameters. (**D**–**I**) Changes due to mating in *w^1118^* and *dSerT^MI02578^* strains in (**D**) total number of interactions, (**E**) in-strength distribution (NOI), (**F**) out-strength distribution (NOI), (**G**) total interaction time, (**H**) in-strength distribution (LOI), and (**I**) out-strength distribution (LOI) as representative social behavior parameters. Statistical comparisons were performed using one-way ANOVA followed by the Holm–Sidak test for normally distributed parameters, or Kruskal–Wallis followed by Dunn’s test for non-normally distributed parameters. ns, not significant. *, **, and *** represent *p* < 0.05, *p* < 0.01, and *p* < 0.001. N = 16–24 flies per condition.

For simplicity, locomotor data are presented here as cumulative distance traveled, and only two motor parameters are shown: mean velocity and activity time (Figure 3A–C). Our results show that virgin *dSerT^MI02578^* female flies traveled a greater cumulative distance than virgin *w^1118^* females, suggesting that chronic impairment of 5-HT signaling results in locomotor hyperactivity (Figure 3A). Interestingly, mating reduced the cumulative distance in *dSerT^MI02578^* females to levels comparable to those of mated *w^1118^* animals (Figure 3A). These results suggest that mating reverses the increased motor output observed in virgin animals with chronically altered 5-HT function. However, examination of specific locomotor parameters reveals that the reversion is incomplete: mated *dSerT^MI02578^* females showed higher mean velocity but lower activity time than mated *w^1118^* females (Figure 3B,C). These data suggest that although overall distance traveled is restored, the underlying movement strategy differs between these strains. Thus, serotonergic impairment affects not only the total locomotor output but also the qualitative organization of the motor program, and mating partially modulates these changes.

When assessing social behavior, virgin *dSerT^MI02578^* females showed fewer fly-to-fly interactions than virgin *w^1118^* females across all parameters measured (total number of interactions, total interaction time, and in-strength and out-strength distribution of interactions by number (NOI) and length (LOI); Figure 3D–I). Strikingly, mating reverted the reduction in fly-to-fly interaction in *dSerT^MI02578^* females, increasing social parameters to levels comparable to those recorded in virgin *w^1118^* animals (Figure 3D–I). Mating had no significant effect on social behavior in *w^1118^* flies. Social network structure parameters were not affected by genotype or mating status (Figure S3A–E), indicating that the topology of the social network is preserved despite changes in interaction frequency and duration. These results indicate that the serotonergic system is required for normal baseline social interactions in virgin females and that mating restores social behavior even when the serotonergic system is chronically impaired.

### 2.4. Responsiveness to Food Odor Is Affected by an Imbalance in the Serotonergic System in Non-Aversive Conditions

**Figure 4:**
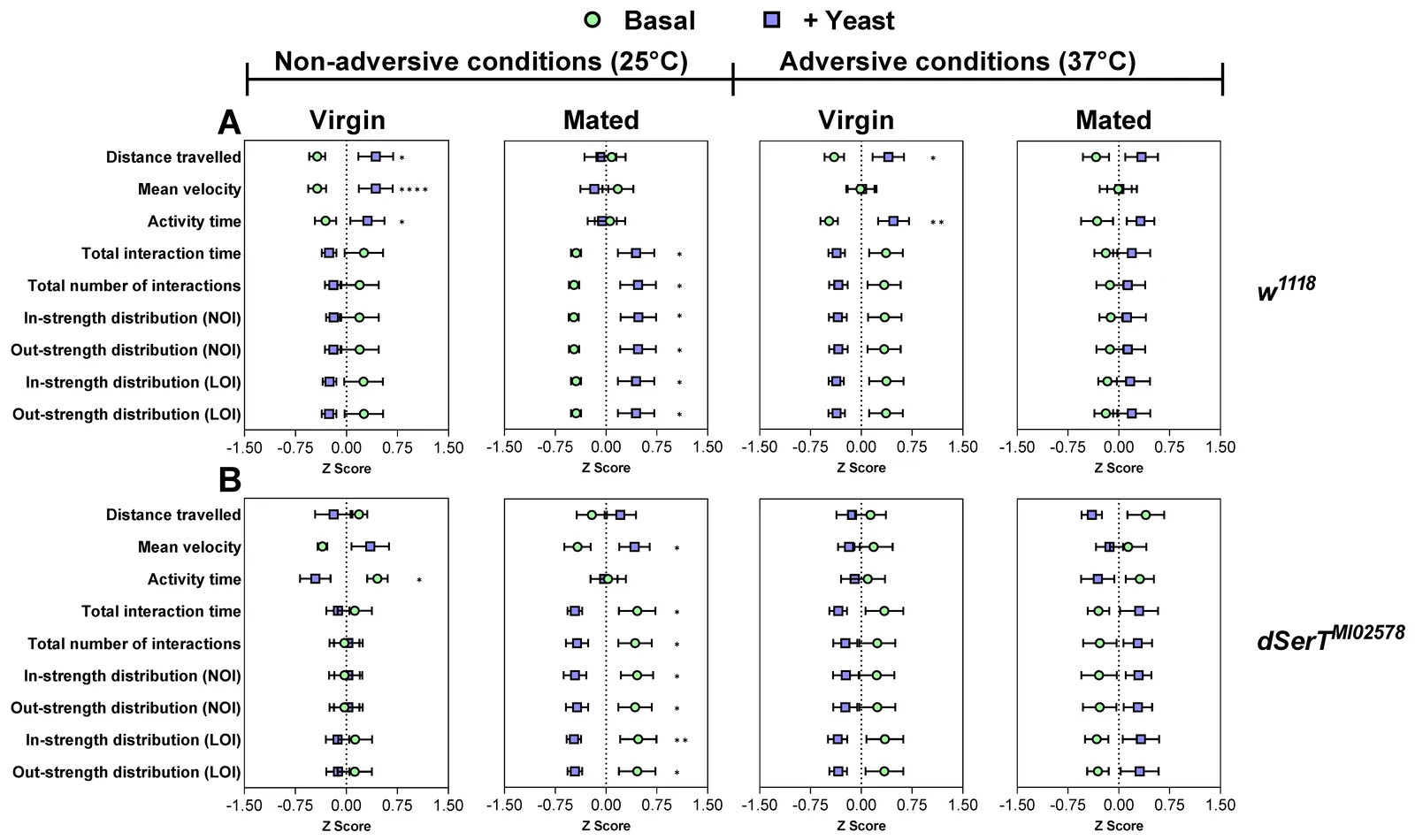
Global serotonergic alteration affects the responsiveness to food odor in non-aversive conditions. Differences between basal and food odor exposure in 9 representative parameters associated with locomotor and social behavior in (**A**) *w^1118^* and (**B**) *dSerT^MI02578^* strains, both virgin and mated, under non-aversive (25 °C) and aversive (37 °C) conditions. Parameter values presented as Z-scores. Statistical analysis carried out using an unpaired *t*-test for normally distributed parameters or Mann–Whitney tests for non-normally distributed parameters with an adjusted *p*-value approach and the Holm–Sidak test with correction for multiple comparisons. *, **, and **** represent *p* < 0.05, *p* < 0.01, and *p* < 0.0001. N = 16–24 flies per condition.

To investigate whether serotonergic signaling influences behavioral responses to food odor, we used the YRT paradigm. Figure 4 shows the data as normalized Z-scores. In *w^1118^* control flies, yeast odor increased locomotion at 25 °C (Figure 4A, left panel), which is similar to what was observed in the CS strain (Figure 2). Mating decreases motor output in *w^1118^* female flies exposed to yeast odor (Figure 4A, second panel), which is overall similar to what was observed in the CS wild-type strain, as well (Figure 2). Interestingly, virgin *dSerT^MI02578^* females show decreased activity time when in the presence of the food odor, while no change was observed in the other motor parameters (Figure 4B, first panel). Additionally, mated *dSerT^MI02578^* females exhibited an increase in mean velocity upon yeast exposure (Figure 4B, second panel), which was not detected in mated *w^1118^* females (Figure 4A, second panel) or virgin *dSerT^MI02578^* females (Figure 4B, first panel). Altogether, these data support the idea that serotonergic impairment alters movement quality in response to food odor rather than broadly affecting locomotion under non-aversive temperature conditions.

We then focused our analysis on social behavior, where the most striking effects were observed. Under non-aversive conditions (25 °C), virgin females of both *w^1118^* and *dSerT^MI02578^* strains showed no changes in social behavior upon yeast exposure (Figure 4A,B, left panels), consistent with what was observed in CS flies (Figure 2). In mated *w^1118^* females, yeast odor increased social interactions across all parameters measured (Figure 4A), recapitulating the response observed in CS flies (Figure 2). Strikingly, mated *dSerT^MI02578^* females showed the opposite response: yeast odor exposure decreased social interactions across all parameters (Figure 4B). This reversal indicates that intact serotonergic signaling is required for the mating-induced increase in social behavior induced by food cues.

Under aversive conditions (37 °C), no changes in social behavior were detected in response to yeast odor in any group, virgin or mated, *w^1118^* or *dSerT^MI02578^* (Figure 4A,B), consistent with the suppression of mating-dependent social responses observed at high temperature in CS flies. Collectively, these results demonstrate that serotonergic signaling is required for the normal social behavioral response to food odor in mated females and that this modulation is specific to non-aversive conditions.

### 2.5. Mating Does Not Alter Whole-Brain 5-HT Levels or Histone Serotonylation in Female Flies

**Figure 5:**
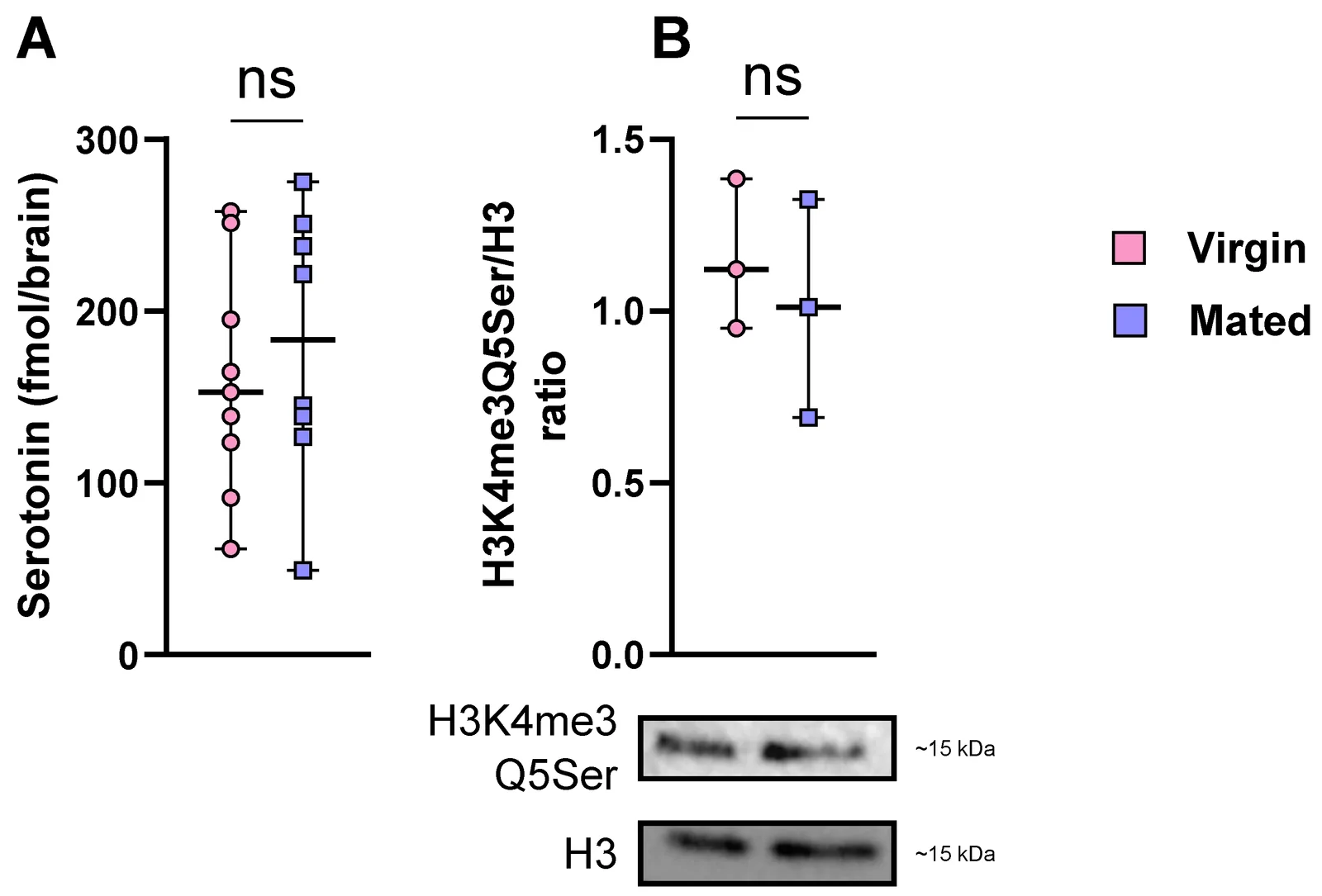
Mating does not alter brain serotonin levels or histone serotonylation in female flies. (**A**) Brain serotonin content measured by HPLC-ECD in virgin and mated CS female flies. Data from N = 8–9 samples, each sample containing 5 fly brains. (**B**). Histone serotonylation levels in brains of virgin and mated CS female strains assessed by Western blot. The ratio of H3K4me3Q5Ser over total H3 is shown. A representative Western blot image is shown below. Statistical comparisons were performed using an unpaired *t*-test. ns, not significant. Data from N = 3 independent samples per experiment; each experiment consisted of 30 female brains.

Our behavioral results indicate that serotonergic signaling plays a key role in modulating post-mating behaviors in flies. To determine whether mating itself alters 5-HT levels in females’ brains, we performed HPLC measurements of brain amine concentrations in virgin and mated females. No significant differences in 5-HT (Figure 5A) or dopamine (DA) levels (Figure S4) were detected between virgin and mated females, suggesting that the post-mating behavioral changes observed in CS flies are not associated with a whole-brain change in biogenic amine content.

Recent studies have shown that 5-HT can be covalently bound to glutamine 5 in histone H3, acting as a permissive epigenetic mark called histone serotonylation [17]. This epigenetic mark is responsible for changes in gene expression that underlie neuronal differentiation [17], synaptic plasticity [24], and circadian behaviors [25] in vertebrate animals. Given that the molecular machinery responsible for writing this epigenetic mark is conserved in flies, including the presence of an ortholog of vertebrate Transglutaminase 2 [26], and that this modification has been previously detected in *Drosophila* larvae [17], together with our behavioral evidence indicating that post-mating responses are modulated by 5-HT, we asked whether histone serotonylation levels are modified by mating. For this, we evaluated H3K4me3Q5Ser levels in fly brain homogenates by Western blot. Our results show that histone serotonylation levels are not different between virgin and mated female flies (Figure 5B). These results suggest that the post-mating behavioral changes are not associated with global changes in brain 5-HT content or 5-HT-dependent chromatin modifications.

### 2.6. The Activity of SPN Neurons Contributes to Changes in Post-Mating Social Behaviors but Not in Locomotion

Since our results indicate that mating does not induce global alterations in either 5-HT levels or histone serotonylation, we proposed that the changes we detected in mated females could be accounted for by specific serotonergic circuits. SPNs are a pair of serotonergic neurons previously associated with post-mating response in *Drosophila*, such as an increase in long-term memory post mating [12], as well as a reduction in female receptivity to mating [15,16]. Thus, we asked whether SPNs contribute to the changes in these behavioral changes.

**Figure 6:**
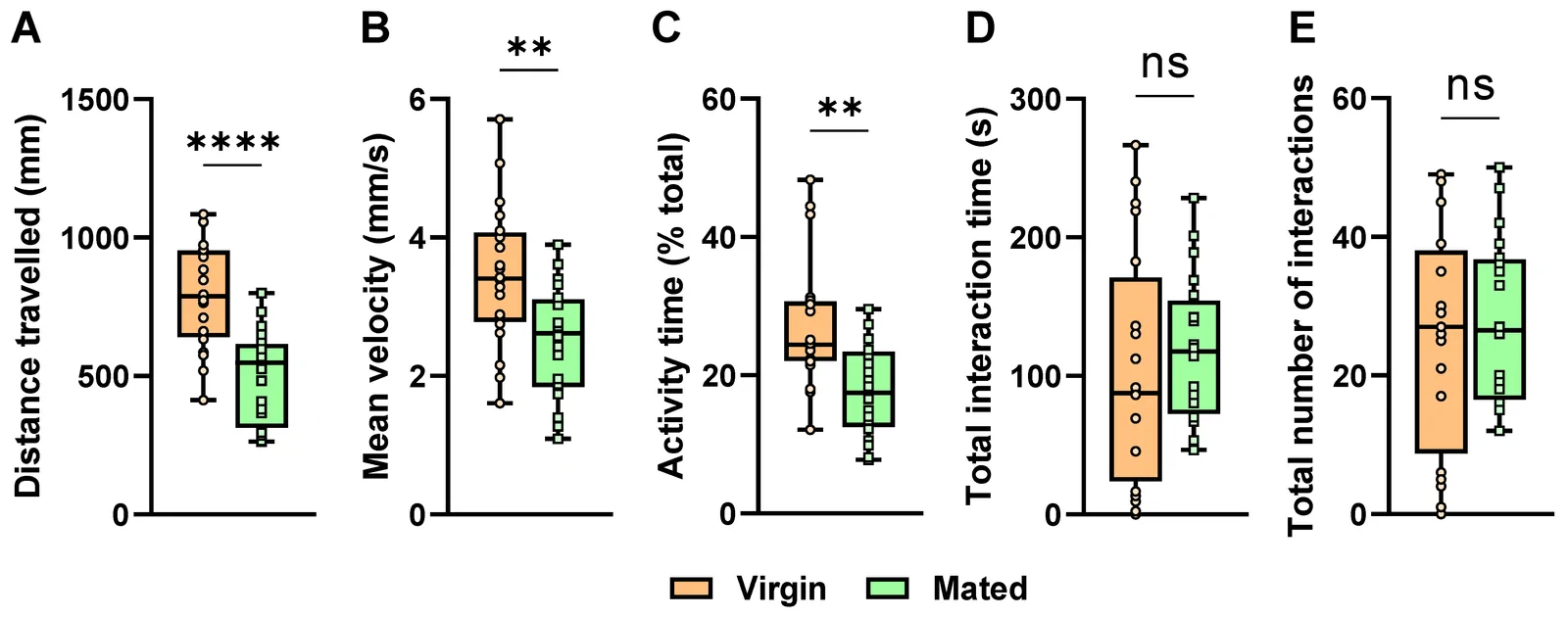
A pair of serotonergic neurons contributes to social parameters but not locomotion. Behavior was video recorded in groups of 5 virgin or mated female SPNSplit > TNT flies. (**A**–**C**) Differences between virgin and mated flies on distance travelled (**A**), mean velocity (**B**), and activity time (**C**), three representative locomotor parameters. (**D**,**E**) Changes due to mating in total interaction time (**D**) and total number of interactions (**E**) as representative social behavior parameters. An unpaired t-test for normally distributed parameters and the Mann–Whitney test for non-normally distributed parameters were used. ns, not significant. ** and **** indicate *p* < 0.01 and *p* < 0.0001, respectively. N = 19–20 flies/condition.

To assess this, we expressed the tetanic toxin light chain (TNT) using a SPN split GAL4 line (SPNsplit > TNT). In these flies, we evaluated the behavior of virgin and mated female flies in basal conditions. Our results indicate that representative locomotor parameters are reduced post-mating in SPNsplit > TNT female flies (Figure 6A–C). These results are consistent with our previous results in CS (Figure 1) and *w^1118^* flies (Figure 2), showing that mating results in an overall reduced locomotion. On the other hand, total interaction time and total number of interactions were not changed due to mating in SPNsplit > TNT flies, which is different compared to our previous results. This would support that these neurons contribute to post-mating changes in social behaviors in female flies. However, the total interaction time and total number of interactions from both virgin and mated SPNsplit > TNT were dramatically increased as compared to CS and *w^1118^* female flies (compare Figure 6D,E with Figure 1G,K, respectively). These results suggest that SPNs contribute to regulating social behaviors but not locomotion.

## 3. Discussion

In this study, we investigated the role of serotonergic signaling in the modulation of locomotor and social behaviors in *Drosophila* females following mating. Our results show that mating reduces locomotor activity and social interactions under control conditions, that exposure to food odor differentially modulates these behaviors depending on the mating status, and that serotonergic signaling is required for the normal expression of mating-dependent social responses to food odor. Importantly, these effects are not accompanied by changes in whole-brain 5-HT content, or histone serotonylation, pointing toward circuit-level mechanisms as the substrate for the 5-HT modulatory role. Consistent with this, we identify the SPNs as a neuronal population that selectively modulates social behaviors in female flies.

### 3.1. Mating Reduces Locomotion and Social Interactions in Female Flies

Our results show that mated females exhibit reduced locomotor activity and fewer social interactions than virgin females, consistent with the idea that mating triggers a shift in behavioral priorities. After copulation, female *Drosophila* transition to a reproductive state focused on securing resources for offspring, including searching for appropriate egg-laying sites and food sources [11,27]. In this context, a reduction in exploratory locomotion and social interactions could reflect an adaptive strategy to conserve energy, minimize unnecessary social engagement, and reduce competition with other females. Interestingly, the reduction in locomotion observed in mated females was characterized by changes in activity-related parameters, such as activity time, walking frequency, and pause frequency, without alterations in speed-related parameters such as mean velocity or acceleration. This suggests that mating mainly affects the motivation to move rather than the locomotor capacity of the animal per se, supporting the idea that mating induces a behavioral program oriented towards energy conservation and risk reduction. The preservation of speed-related parameters suggests that mated females maintain intact motor capacity, which could enable them to rapidly increase locomotion when required, for instance, when facing a threat.

### 3.2. Food Odor Exposure Reveals Mating-Dependent Differences in Social Behavior

To uncover new aspects of post-mating behavioral changes in female flies, we developed the YRT paradigm, a simple and effective tool for studying state-dependent behavioral responses in grouped animals, which may be useful for future studies investigating the neural and molecular mechanisms underlying food-seeking behavior in the context of the reproductive state.

A key finding of this study is that exposure to yeast odor, a naturalistic food cue, reveals behavioral differences between virgin and mated females that are not evident under basal conditions. Specifically, only mated females increased their social interactions in response to food odor under non-aversive conditions. This finding suggests that mating primes females to respond to food-related cues by increasing social engagement around potential food sources, possibly as an adaptive strategy to support offspring development. This interpretation is consistent with previous reports showing that mated females exhibit a stronger preference for protein-rich food sources such as yeast [28,29] and extends those observations by demonstrating that this post-mating shift also includes a social component. An alternative explanation is that the increased social interactions observed in mated females exposed to yeast odor reflect competitive encounters over food resources rather than cooperative aggregation. Such competition might be particularly relevant after mating, when nutritional demands increase to support egg production and offspring development [30]. Distinguishing between cooperative and competitive interactions would require higher-resolution behavioral analyses in future studies.

We then carried out behavioral studies at 37 °C. This temperature was selected to generate a strong aversive context, allowing the assessment of how competing environmental and physiological signals shape behavioral responses. The suppression of the mating-dependent responses under the aversive temperature further supports the idea that the behavioral changes observed are not simply a consequence of altered sensory processing but rather reflect a state-dependent integration of environmental and physiological signals. The fact that virgin females largely maintained their locomotor response to yeast odor at high temperature while mated females did not suggests that the motivational weight of food-related cues differs between reproductive states and is sensitive to competing aversive signals.

### 3.3. 5-HT Modulates Mating-Dependent Social Responses to Food Odor

The use of the *dSerT^MI02578^* hypomorphic mutant revealed that chronic alteration of 5-HT transport activity, which results in long-term hindering of serotonergic signaling, has distinct consequences depending on the mating status and environmental context. Virgin *dSerT^MI02578^* females exhibited locomotor hyperactivity and showed reduced social interactions compared to control virgin *w^1118^* females, consistent with a role for 5-HT in suppressing locomotion and facilitating social engagement under basal conditions. These findings are in line with previous reports showing that serotonergic signaling regulates locomotor activity [20,21,31] and social behavior [32] in *Drosophila*.

Interestingly, mating reverted both the locomotor hyperactivity and the social interaction deficit in *dSerT^MI02578^* females, suggesting that mating-associated physiological changes can compensate for chronic serotonergic impairment under basal conditions. However, this reversion was partial: although overall distance traveled was restored, mated *dSerT^MI02578^* females showed higher mean velocity but lower activity time than mated control females, indicating that the qualitative organization of motor programs remained altered. This dissociation between global locomotor output and movement quality suggests that 5-HT contributes to the fine-tuning of motor strategies rather than simply gating overall activity levels.

Most strikingly, when *dSerT^MI02578^* mated females were exposed to yeast odor in the YRT, their social behavioral response was inverted relative to control females: while mated *w^1118^* females increased social interactions in response to food odor, mated *dSerT^MI02578^* females decreased them. This reversal was specific to non-aversive conditions, as no social behavioral changes were detected in response to yeast odor at 37 °C in either strain. Overall, these results indicate that intact serotonergic signaling is required not only for normal baseline social behavior but also for the appropriate modulation of social responses by food-related environmental cues in the context of the post-mating state. Thus, our results support that this monoaminergic system may act as a critical integrator of the internal physiological state and external sensory information to generate appropriate social behavioral responses.

One limitation of our study is the use of a single mutant that chronically affects the serotonergic system, *dSerT^MI02578^*. On the other hand, chronic impairment of 5-HT transporter function from early development could trigger compensatory changes, including homeostatic adjustments in serotonin receptor expression, altered wiring or branching of serotonergic circuits during development, or upregulation of other monoaminergic components. Hence, the behavioral phenotypes we report may reflect a combination of the direct loss of 5-HT reuptake and these potential secondary developmental adaptations. Future studies should employ other tools to globally and timely affect the operation of the serotonergic system to confirm our findings.

### 3.4. Mating Does Not Alter Global 5-HT Levels or 5-HT-Dependent Epigenetic Marks

The literature shows that several behaviors are affected by 5-HT in mated female flies [15,16]. The data presented here demonstrate a prominent role of 5-HT in modulating several additional behaviors in female flies after mating. Thus, we hypothesized that a global change in the operation of several serotonergic circuits might explain the effects of this amine on a wide range of behaviors in the brain of mated females. However, our HPLC measurements revealed no significant differences in total brain 5-HT content between virgin and mated females. Similarly, no changes in histone serotonylation were detected, arguing against a role for 5-HT-dependent chromatin remodeling in the regulation of post-mating behavior. The absence of changes in dopamine levels further argues against a whole-brain level reorganization of monoaminergic signaling following mating.

### 3.5. SPNs Are Required for Social but Not Locomotor Changes Induced by Mating

These negative results have important mechanistic implications, as they support that the contribution of 5-HT to post-mating behavior is not mediated by global brain changes in neuromodulator availability but rather by changes in the activity or connectivity of one or a few specific circuits, in the sensitivity of postsynaptic 5-HT receptors, or some other mechanism. This interpretation is consistent with the emerging view that neuromodulatory systems exert state-dependent effects through circuit-specific mechanisms rather than global changes in neuromodulator tone [33]. In this regard, a particularly interesting circuit is that constituted by SPNs. These are a pair of serotonergic neurons that express the sex peptide receptor, are activated following mating, and have been shown to modulate long-term olfactory memory in female flies after mating [12,34], as well as being part of the changes in female receptivity to new males after mating [16].

To test the role of SPNs, we inhibited synaptic function in these neurons using TNT and studied behavioral features in virgin and mated female flies in this strain. Our results show that, after mating, it is still possible to detect the reduction in locomotor parameters in SPNsplit > TNT females, mirroring the behavioral pattern observed in CS and *w^1118^* flies. This indicates that SPN activity is dispensable for the mating-induced reduction in locomotion and suggests that a distinct serotonergic circuit, one that remains functionally intact despite SPN silencing, might mediate this locomotor change dependent on mating status. In contrast, total interaction time and total number of interactions no longer changed after mating in SPNsplit > TNT flies, which is different from the robust reduction observed in CS females. This data supports that SPN activity is selectively required for the social but not the locomotor component of the post-mating behavioral response. Interestingly, social behavior in these flies is dramatically increased, which supports that the SPNs are involved in basal social behavior in flies, and not only in mating-induced changes. In this regard, it should be emphasized that these results do not rule out contributions from other serotonergic neurons, downstream postsynaptic targets, or parallel circuits acting in concert with SPNs to modify social behavior in female flies. Our data should be interpreted as identifying one necessary node rather than the full mechanistic pathway.

Overall, our results support that 5-HT plays a key role in female flies, modulating motor and social behavioral responses to food odor in a mating-dependent manner. This modulation is due to changes in specific and discrete serotonergic circuits, such as those involving SPNs, rather than global changes in 5-HT.

## 4. Materials and Methods

### 4.1. Fly Stocks and Mating Protocol

The *w^1118^* (RRID:BDSC_6326); *dSerT^MI02578^* (y[1]w[*];Mi{y[+mDint2]=MIC}SerT[MI02578]; RRID:BDSC_36004); SPNsplit-GAL4 (w[1118]; P{y[+t7.7] w[+mC]=R61A01-p65.AD}attP40; P{y[+t7.7] w[+mC]=VT057280-GAL4.DBD}attP2; RRID:BDSC_75829); and UAS-TNT (w[*]; P{w[+mC]=UAS-TeTxLC.tnt}R3; RRID:BDSC_28997) fly strains were obtained from the Bloomington *Drosophila* Stock Center (Bloomington, IN, USA). The CS stock was already available in the Campusano lab. *dSerT^MI02578^* flies were backcrossed into the *w^1118^* genetic background for at least 5 generations. Flies were raised at 25 °C in a 12 h light/dark cycle in standard food vials. Virgin female flies were separated at day 0 post-hatching and maintained with at least two more female flies to avoid the effects of social isolation. For the mated group, an excess of male flies was introduced to the vial containing female flies on day two post-hatching and removed during day 3 to ensure mating of the female flies. Female flies were tested on their behavior on days 4 to 7 post-hatching, that is, 1–3 days post-mating.

### 4.2. Behavioral Setup

Female flies were anesthetized on ice and separated into groups of 4–6 individuals. They were maintained over a white acrylic plate and covered with a 35 mm petri dish lid (39 mm diameter). All behavioral tests were carried out in the fly behavior room, which was maintained at 23 °C and 40–60% humidity. All experiments were performed between ZT2 and ZT4 to control for circadian effects. Animals were allowed to acclimate for at least 40 min before the behavioral tests began.

The behavioral arena consists of an acrylic plate covered with a petri dish lid inside a white box with no visual information that could bias fly behavior. This setup is placed on a thermal plate set at 25 °C. Whenever required, the thermal plate was set at 37 °C for the duration of the recording period (5 min). This is considered a robust aversive, stressful condition in *Drosophila* [18,19]. Fly activity was video recorded with a Basler acA2500 camera using pylon Viewer 7.4.0.14100 (Basler AG, Ahrensburg, Germany). The camera was placed above the arena (as shown in the general arrangement in Figure 2). The recorded videos (5 min each) were captured at 1280 × 1080 pixels.

The petri dish lids have two small holes at opposite sides. The size of the holes prevents flies from escaping the arena but allows external odors to enter. We placed cotton bars outside the arena, next to the holes, and secured them with molding clay. A total of 100 µL of water or yeast solution was applied or not to the cotton bars, depending on the experiment. Video recordings were obtained of groups of flies before and during exposure to the food/attractive odor.

For the preparation of liquid yeast, we used a modified version of a protocol previously reported [30]. Briefly, 100 mL of apple juice was heated to 70–75 °C under agitation, then supplemented with 2.5 g of sucrose and 0.25 g of agarose. While the mix is still hot, 2.5 g of bread yeast was added. This preparation was mixed until it looked clear.

### 4.3. Measurement of Whole-Brain 5-HT and DA Content

We followed a general protocol previously reported [35,36]. Endogenous 5-HT and DA levels were quantified by high-performance liquid chromatography (HPLC) coupled with electrochemical detection (HPLC-ECD). For this, 5 μL of the samples was injected into a reverse-phase HPLC. The column used was an Inertsil ODS-3 3 μm 3.0 I.D. × 100 mm (GL Sciences 5020-04424, Tokyo, Japan). The HPLC mobile phase consisted of acetonitrile 8%, 0.1 M sodium phosphate monobasic, 1.8 mM 1-octane sulfonic acid, and 1 mM EDTA, at pH 3.0, and it was pumped at 0.5 mL/min. Electrochemical detection was performed using an amperometric detector (LC-4C, Bioanalytical Systems Inc., BASi, West Lafayette, IN, USA) equipped with a thin-layer analytical cell, a glassy carbon working electrode, and an Ag/AgCl reference electrode, with the working electrode potential set at +0.650 V. In these conditions, the retention times for DA and 5-HT were approximately 4.5 min and 13 min, respectively. Quantification was performed by comparing the peak area of 5-HT or DA in the 5 μL injected sample against calibration curves built from serial dilutions of 5-HT and DA standards (ranging from 15.625 to 250 fmol, R^2^ ≥ 0.99 for both analytes). The limit of detection (LOD) and limit of quantification (LOQ) were calculated as 3.3 × SD and 10 × SD of the baseline noise (N = 8 blank injections), respectively, divided by the slope of the calibration curve. LOD/LOQ values were 4.84/14.7 fmol for DA and 0.85/2.6 fmol for 5-HT.

### 4.4. Western Blot for Histone Serotonylation

Samples were obtained from 30 *Drosophila* adult brains homogenized in RIPA buffer (10 mM Tris-HCl pH 8.0, 1 mM EDTA, 1% Triton X-100, 0.1% sodium deoxycholate, 0.1% SDS, 140 mM NaCl) supplemented with proteinase inhibitors (Pepstatin 1×, Leupeptine 1×, Benzamidine 1×, Antipaine 1×, PMSF 1×). Homogenized samples (10 μg of total protein) were heated at 95 °C for 10 min and then maintained on ice for at least 5 min. After this, proteins were separated by SDS-PAGE. Proteins were transferred to 0.2 μm PVDF membranes at 300 mA for 3 h. Transferred membranes were blocked with 5% milk TBS 1× 0.1% Tween-20 (TBS-T) for 1 h at room temperature. After 3 rinses with TBS-T, membranes were incubated with rabbit anti-H3K4me3Q5Ser (Cat No. AB2580, Sigma-Aldrich, St. Louis, MO, USA) (1:1000) for 2 days. Afterwards, membranes were rinsed and then incubated with goat anti-rabbit HRP (Cat No. AB6721, Abcam, Cambridge, UK) (1:5000) secondary antibody for 2.5 h at room temperature. Positive signals were revealed using Westar Supernova (XLS3, Cyanagen, Bologna, Italy). For H3 measurement as loading control, membranes were stripped using 1× Re-Blot Plus Strong Solution (Cat No. 2504, Merck, Darmstadt, Germany) for 25 min after H3K4me3Q5Ser measurement. Primary antibody anti-H3 (Cat No. AB1791, Abcam) (1:10,000) was incubated overnight, and the protocol for secondary antibody incubation was repeated. Detection of anti-H3 positive signals was performed using Westar Sun (XLS063, Cyanagen).

### 4.5. Data Extraction, Quantification, and Statistical Analysis

The video recordings were analyzed using a modified data extraction from the IowaFLI Tracker software (version 3.1) [37]. We obtained X and Y coordinates of every fly in the video in an .xlsx file. Then, we used a custom Python (version 3.12.9) script developed in our laboratory to obtain all locomotor parameters and preference indices. We also estimated the orientation of each fly in each frame as the angle between its position in the current frame and its position in the previous frame, using arctan2 (X, Y). To calculate social network parameters, we used the orientation and positional information of every fly in the video based on the NetworkX Python library (version 3.1) using a script developed by Petrović et al. (2023) [38]. We considered that, as an interaction criterion, two flies must be at a maximum distance of 3 mm from each other and that this distance must be maintained for at least 2 s. The angle formed between two flies to be considered an interaction must be between −90° and 90°.

For some sets of results, Z-score normalization and standardization were used. The Z-score is calculated by subtracting the mean of the corresponding experimental group from each individual value and then dividing the number obtained by the group’s standard deviation.

The resulting data were plotted and analyzed using GraphPad Prism 9 software (GraphPad Software, San Diego, CA, USA). Detailed information about statistical tests used in experiments is provided in the figure legends. We used Gephi software (version 0.10.1) to obtain social network graphs.

## Data Availability

The original contributions presented in this study are included in the article/Supplementary Materials. Further inquiries can be directed to the corresponding author(s).

## Supplementary Materials

The following supporting information can be downloaded at https://www.mdpi.com/article/10.3390/ijms27167070/s1.

## Abbreviations

The following abbreviations are used in this manuscript:

5-HT	Serotonin
CS	Canton-S
DA	Dopamine
dSerT	*Drosophila* Serotonin transporter
HPLC	High-performance liquid chromatography
HPLC-ECD	High-performance liquid chromatography coupled with electrochemical detection
LOI	Length of interactions
NOI	Number of interactions
SPNs	Serotonin projection neurons
TBS-T	TBS-Tween-20 solution
TNT	Tetanic toxin light chain
YRT	Yeast-response test
