# Supplementary material for: Mating-Induced Behavioral Changes in Female Drosophila Are Modulated by Serotonin
Source: Int J Mol Sci. 2026 Aug 7;27(16):7070. doi: 10.3390/ijms27167070 (PMC13512695; doi:10.3390/ijms27167070)
Supplement: Supplementary file 1 [file ijms-27-07070-s001.zip › ijms-4324896-supplementary.pdf]

SUPPLEMENTARY INFORMATION

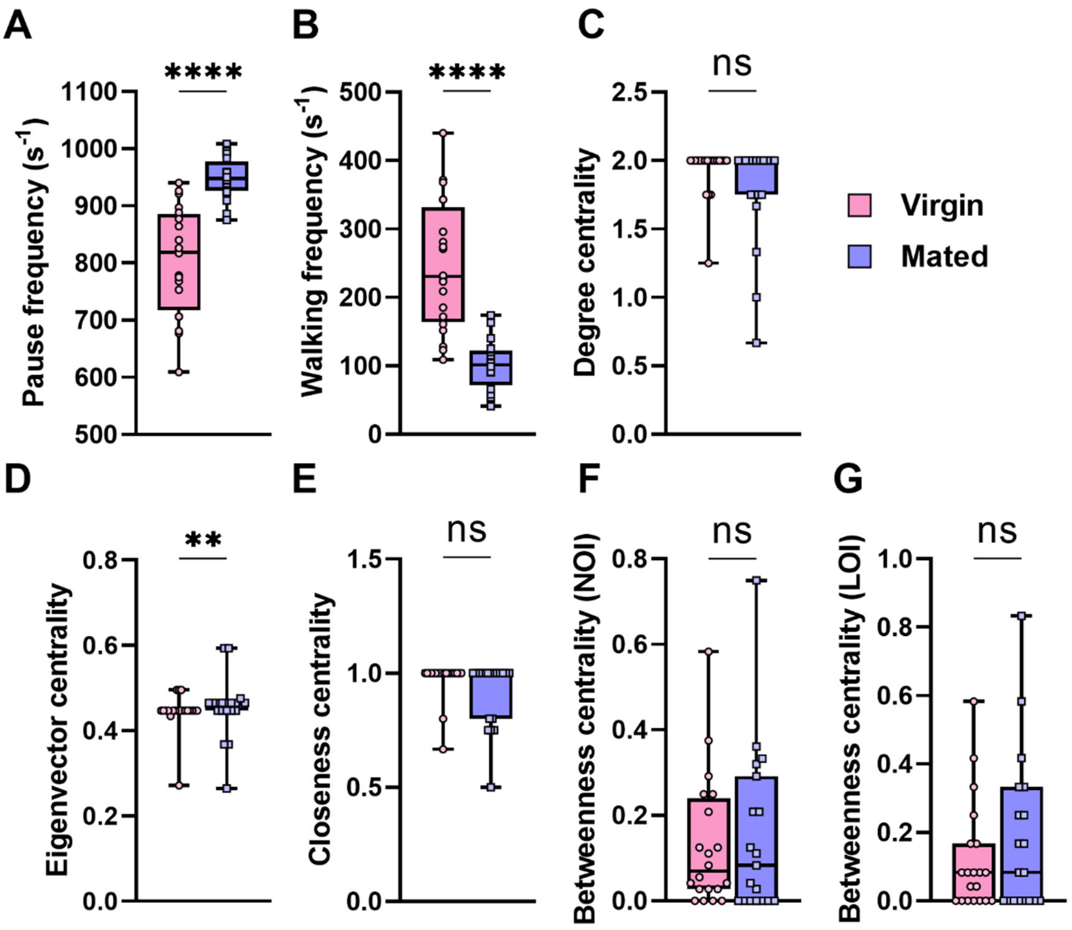

Figure S1: Mating affects parameters defining motor programs in female flies. Behavior was video recorded in groups of 4-6 virgin or mated female flies of the CS strain. Their locomotor and fly-to-fly social behaviors were analyzed. Locomotor behavior was recorded in virgin and mated female flies and assessed in several parameters: (**A**). pause frequency, (**B**). walking frequency. **(C-G)** Representative parameters that serve to characterize social network structure in virgin and mated female flies: (**C**)**.** degree centrality, (**D**). eigenvector centrality, (**E**). closeness centrality, (**F**). betweenness centrality (NOI), and (**G**)**.** betweenness centrality (LOI). Statistical comparisons were performed using the unpaired t-test for normally distributed parameters, and the Mann-Whitney test for non-normally distributed parameters. ** and **** represent p<0.01 and p<0.0001, respectively, while “ns” indicates no significant differences.

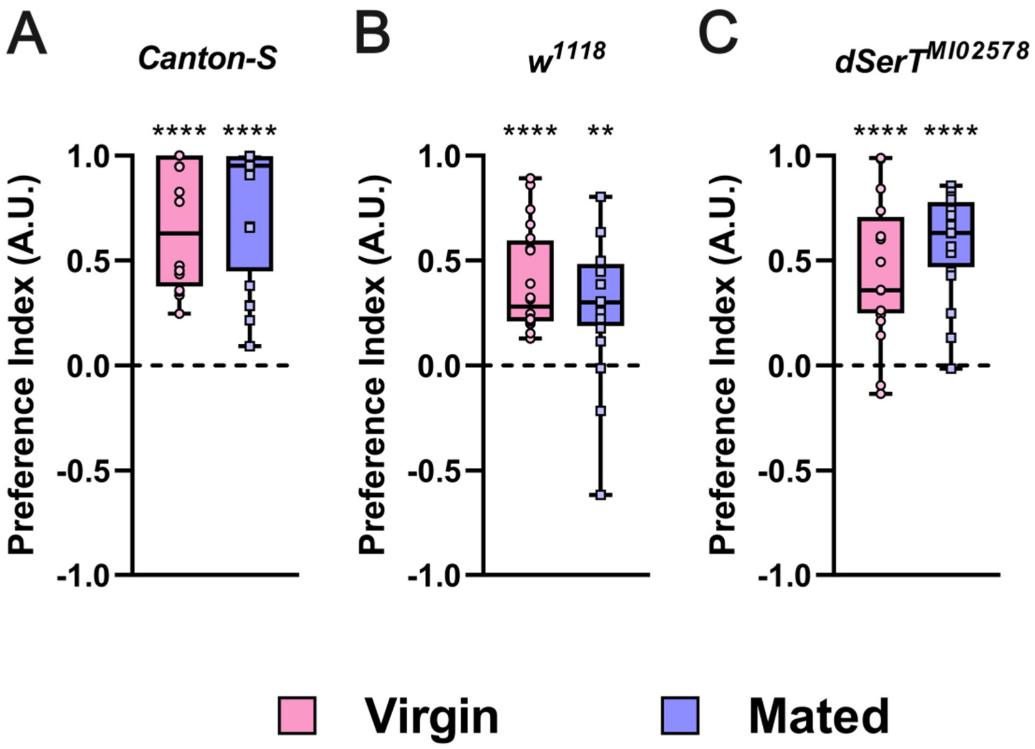

Figure S2: Female flies are able to respond to the yeast odor regardless of the reproductive state. Female flies of the (A). *Canton-S*, (B). w^{1118}, and **(C).** *dSerT*^{MI02578} strains were exposed to yeast odor. Behavior was video recorded and results are expressed as Preference Index (in arbitrary units, A.U.), where 1.0 represents full attraction, while -1.0 indicates full repulsion. Differences in the preference index relative to the no odor/no-preference index (0.0) are shown for the strains used in this study. Statistical comparisons were performed using a one-sample Wilcoxon test. ** and **** represent $p<0.01$ and $p<0.0001$.

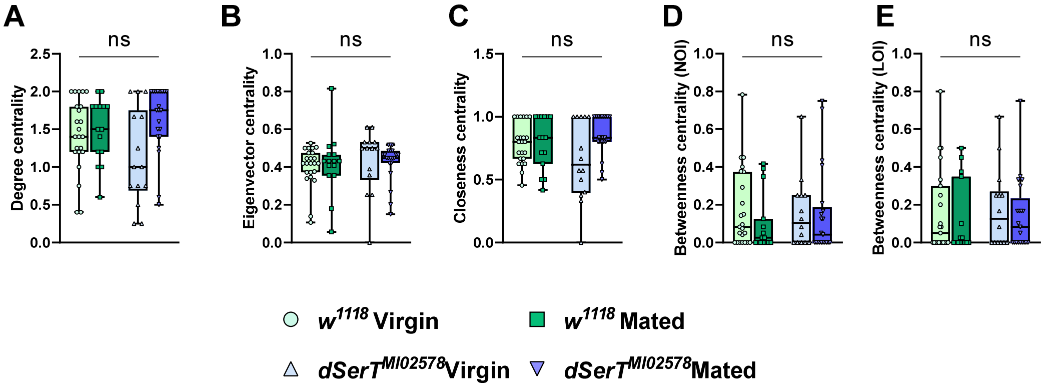

Figure S3: Chronic impairment in serotonergic signaling does not affect social network parameters. Results obtained for virgin and mated female flies of the *w*^{1118} and *dSerT*^{MI02578} strains on social network parameters: **(A).** degree centrality, **(B).** eigenvector centrality, **(C).** closeness centrality, **(D).** betweenness centrality (NOI), **(E)** betweenness centrality (LOI). Statistical comparisons were performed using a One-way ANOVA, followed by the Holm-Sidak test for normally distributed data, or the Kruskal-Wallis test, followed by Dunn's test for non-normally distributed data. No significant differences were found between groups (ns).

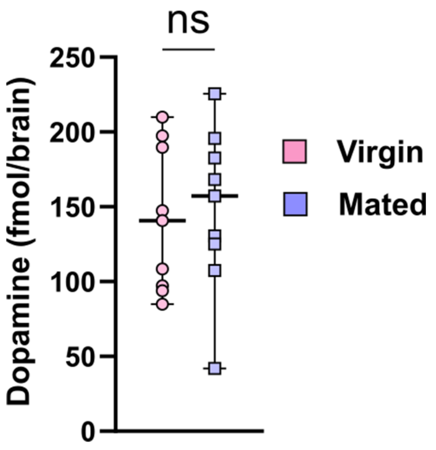

Figure S4: Mating does not change brain dopamine levels in female flies. Brain dopamine content measured by HPLC-ECD in virgin and mated *CS* female flies, expressed as fmol per brain. Statistical comparisons were performed using an unpaired t-test. ns, not significant differences.
